# SELDI-TOF-MS ProteinChip array profiling of T-cell clones propagated in long-term culture identifies human profilin-1 as a potential bio-marker of immunosenescence

**DOI:** 10.1186/1477-5956-5-7

**Published:** 2007-06-05

**Authors:** Dawn J Mazzatti, Graham Pawelec, Robin Longdin, Jonathan R Powell, Rosalyn J Forsey

**Affiliations:** 1 Unilever Corporate Research, Colworth Park, Sharnbrook, UK; 2 Zentrum für Medizinische Forschung, Universitätsklinikum Tübingen, Tübingen, Germany; 3 LCG Biosciences, Bourn, UK

## Abstract

**Background:**

The adaptive immune response requires waves of T-cell clonal expansion on contact with pathogen and elimination after clearance of the source of antigen. However, lifelong persistent infections with common viruses cause chronic antigenic stimulation which takes its toll on adaptive immunity in late life. Chronic antigenic stress results in deregulation of the T-cell response and accumulation of anergic cells. Longitudinal studies of the elderly show that this impacts on survival. Identifying the nature of the defects in chronically-stimulated T-cells and protein bio-markers of these dysfunctional cells would help to understand age-associated compromised T-cell function (immunosenescence) and facilitate the development of targeted intervention strategies.

The purpose of this work was to use surface-enhanced laser desorption/ionization time-of-flight mass spectrometry (SELDI-TOF-MS) to analyse proteins associated with T-cell senescence in order to identify potential bio-markers. Clonal populations of T-cells isolated from elderly octogenarian and centenarian donors were grown *in vitro *until senescence, and early passage and late passage (pre-senescent) cells were analysed using SELDI-TOF-MS ProteinChip arrays.

**Results:**

Discriminant analysis identified several protein or peptide peaks in the region of 14.5–16.5 kDa that were associated with T-cell clone senescence. Human profilin-1, a ubiquitous protein associated with actin remodelling and cellular motility was unambiguously identified. Altered expression of profilin-1 in senescent T-cell clones was confirmed by Western blot analysis.

**Conclusion:**

Due to the proposed roles of profilin-1 in cellular survival, cytoskeleton remodelling, motility, and proliferation, it is hypothesised that differential expression of profilin-1 in ageing may contribute directly to immunosenescence.

## Background

Immunosenescence – or dysfunction of the immune response that occurs during ageing-- contributes significantly to increased morbidity and mortality in the elderly population [1-3]. Dysfunctional immunity in ageing is particularly prevalent in the T-cell compartment; T-cells derived from elderly individuals show poor proliferative responses, clonal expansion and accumulation of memory and effector T-cells, and the exhaustion of naïve T-cells. Immunosenescent T-cells also exhibit dysregulated signalling pathways in addition to altered adhesion/activation molecule expression and production of cytokines, ultimately resulting in phenotypic and functional alterations to T-cell mediated immunity [2,4-7].

In humans, cytomegalovirus (CMV), which is prevalent in the elderly population, contributes markedly to the persistent clonal expansion of CD8 T-cells observed in ageing [8-10]. In fact, an immune risk phenotype (IRP) has been described encompassing bio-markers associated with the age-related decline in immune function predictive of mortality in longitudinal studies [11]. One of the major characteristic features of human immunosenscence *in vivo *is the predominance of clonal expansions of a limited repertoire of CD8+/CD28- cells [9,12,13]. The majority of CD28 negative T-cells can produce pro-inflammatory cytokines [14] but in the very elderly these cells are further compromised in the production of all cytokines on antigen-specific challenge [15]. Individuals with the IRP also exhibit a pro-inflammatory phenotype, providing further evidence of age related alterations in both innate and adaptive immune systems [16]. These dysregulated, immunosenescent T-cells fill up the 'immunological space' resulting in a general immuno-suppression through lack of provision of secondary activating signals – adhesion/activation and soluble mediators.

While attempts have been made to characterize the phenotypic changes that occur during T-cell ageing and immune dysregulation, the mechanistic drivers underlying T-cell immunosenescence are poorly understood. At the cellular level, age-related loss of immune function is associated with the accumulation of cells with (1) decreased membrane fluidity and calcium influx [1] and (2) signal transduction defects, particularly in the cellular stress [7] and redox regulatory pathways (reviewed in [17]). Importantly, regulation of the redox-sensitive transcription factor NFκB is altered in T-cell senescence [18] and may impact on several different down-stream signalling targets. These data suggest that global regulatory and signalling networks may be altered in dysfunctional, ageing T-cells. Understanding the mechanistic drivers that lead to the accumulation of dysfunctional cells may allow the development of an intervention in the elderly to reconstitute appropriate immune responses. To this end, *in vitro *T-cell models of clonal expansion provide useful tools, enabling the production of adequate sample material to investigate the complex mechanistic drivers of T-cell ageing using 'omic' technologies. Using this *in vitro *system to guide the discovery of protein bio-markers of ageing and immunosenescence *in vivo *could aid in understanding immune dysfunction in the elderly and would allow development of novel intervention strategies to restore function [19,20].

The current study utilised surface enhanced laser desorption/ionisation time-of-flight mass spectrometry (SELDI-TOF-MS) ProteinChip Array technology (Ciphergen Biosystems, Inc., Fremont, CA) for protein profiling of ageing and senescent T-cells. This method is unique in that it uses chromatographic surfaces to retain proteins based on their physicochemical characteristics, followed by TOF-MS using a ProteinChip Reader (PBSIIc Series 4000; Ciphergen Biosystems, Inc.). Isolated proteins are separated on different array surfaces including cation/anion-exchangers, hydrophobic and metal-ion affinity surfaces. Proteins that bind and are retained on the surfaces are analysed by mass spectrometry (MS). This technique offers enhanced sensitivity which is ideal for the analysis of small sample volumes and allows screening of low-molecular weight proteins. The SELDI-TOF/MS approach has several advantages for bio-marker analyses. Firstly, it requires little starting material; secondly, it has a high-throughput capacity; thirdly it allows semi-quantitative and statistical analysis; and finally, it can aid in the identification of purification conditions prior to protein identification by peptide mass fingerprinting. As such, SELDI technology has been applied in recent years to investigate a variety of biological phenomena, due to the increasing need for high-throughput technology that allows the detection, purification, and identification of proteins at high speed and with high sensitivity [21,22].

The main objectives of the current study were to use SELDI-TOF-MS to profile proteins that were differentially expressed in chronic antigenic stress-induced T-cell clone senescence. Protein profiles from T-lymphocytes isolated from elderly donors grown to pre-senescence in culture were compared to early passage clones in order to identify proteins associated with *in vitro *ageing and senescence. Using SELDI-TOF-MS analysis, we identified several candidate protein or peptide peaks that were preferentially expressed in late passage T-cell clones. Differential expression of one protein, human profilin-1, was confirmed by Western blot analysis comparing early and late passage T-cell clones. Human profilin-1 is thought to be involved in cytoskeleton remodelling, adhesion, and proliferation and migration [23-26], all of which are cellular processes that may impact on immunosenescence. As such, profilin-1 may represent a potential bio-marker associated with T-cell dysfunction and development of senescence.

## Results

### Identification of protein/peptide peaks by SELDI-TOF-MS

**Figure 1:**
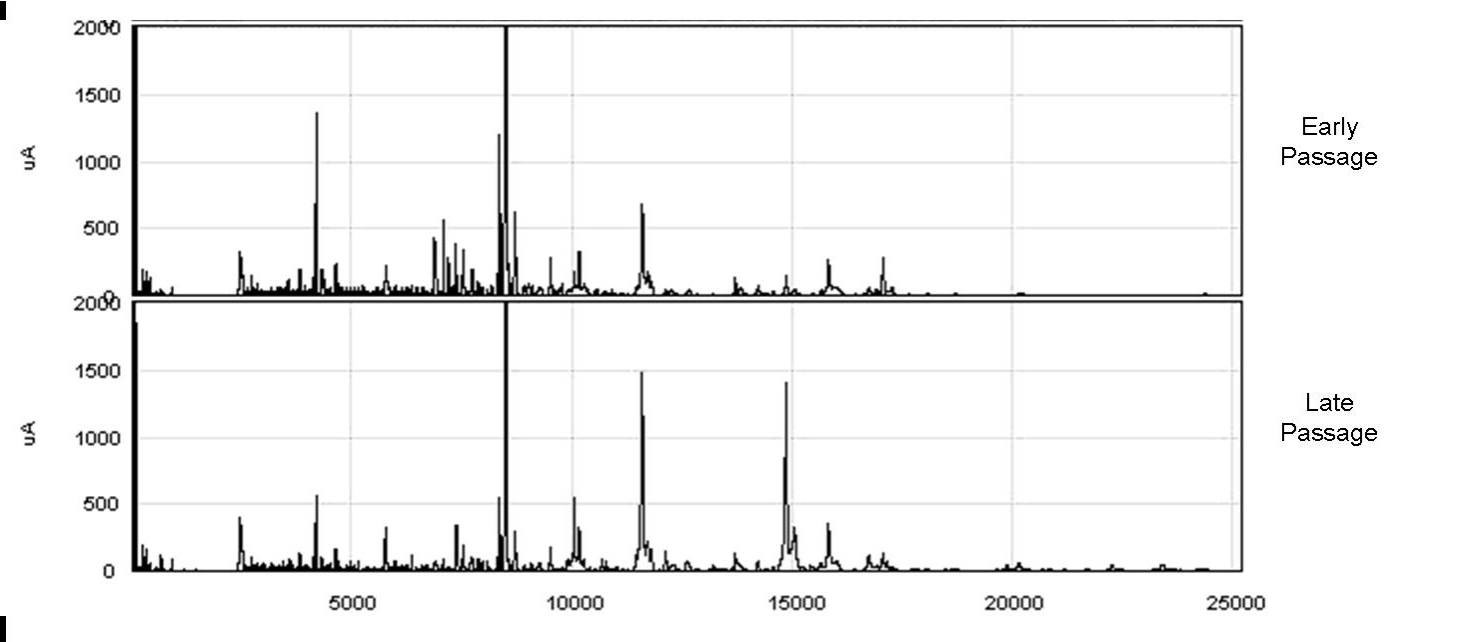
**Surface-enhanced laser desorption/ionisation profiles of early and late passage T-cell clones derived from octogenarian donors**. CD4+ human T-cell clones from activated peripheral blood lymphocytes of healthy octogenarian donors were obtained by limiting dilution in the presence of IL-2. Protein profiling was assessed at timepoints as early as possible in antigen-stimulated *in vitro *culture (at the first population doubling (PD) in which two million cells were available for analysis) and approaching replicative senescence (at the penultimate or final PD before clones ceased to replicate). SELDI-TOF-MS analysis was performed using H50 reversed-phase ProteinChips. Spectra shown are the representative of early passage and late-passage T-cell clones. Normalised mass (m/z) for each peak (in Daltons (Da)) is demonstrated on the X-axis, while intensity is plotted on the Y-axis.

In order to determine which proteins are differentially expressed as cells approach senescence, early and late passage T-cell clones were analyzed on two different ProteinChip array chemistries as described in *Materials and Methods*. Samples were grouped based on passage time and expression difference mapping (EDM) was utilised (Ciphergen Express™ software). Peaks were assigned in each sample and those which significantly differed (p < 0.05) between early and late passage T-cell clones were determined. Figure 1 shows representative spectra obtained from early and late passage samples.

**Figure 2:**
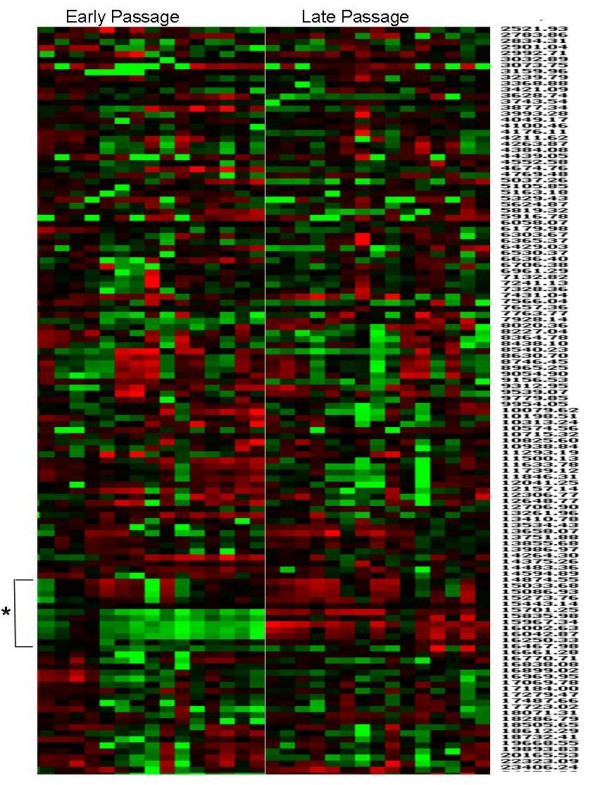
**Heat map visualisation of early and late passage T-cell clones**. Data obtained from SELDI analysis of early and late passage CD4+ human T-cell clones using H50 reversed-phase ProteinChips were visualised by clustering and heat map analysis. A region between 14.5 kDa and 16.5 kDa, marked by asterisk, was identified to be highly conserved between individual samples and significantly differed between early and late passage T-cell clones.

EDM averages the intensities of each peak for each sample in the group, in order to determine whether the mean intensity differs between two groups. As such, in this approach there is no consideration of how many samples within a group exhibit the trend, and the result is influenced strongly by outlying samples. In order to visualise areas of the proteome that differed in the majority of samples in one group compared to the majority of samples in the other group, hierarchical clustering was performed and visualised by heat map, as shown in Figure 2. A heat map is a graphical representation of the entire array in grid form, with columns representing samples and rows representing the m/z of protein/peptide peaks. The intersection of a peak and sample is coloured according to its expression value compared to the mean of the entire sample set. In this analysis red indicates high expression compared to the mean while green indicates low expression compared to the mean intensity value. Black colouring indicates no change from the mean. In addition the intensity of the colour indicates the extent of over- or under-expression compared to the mean.

**Figure 3:**
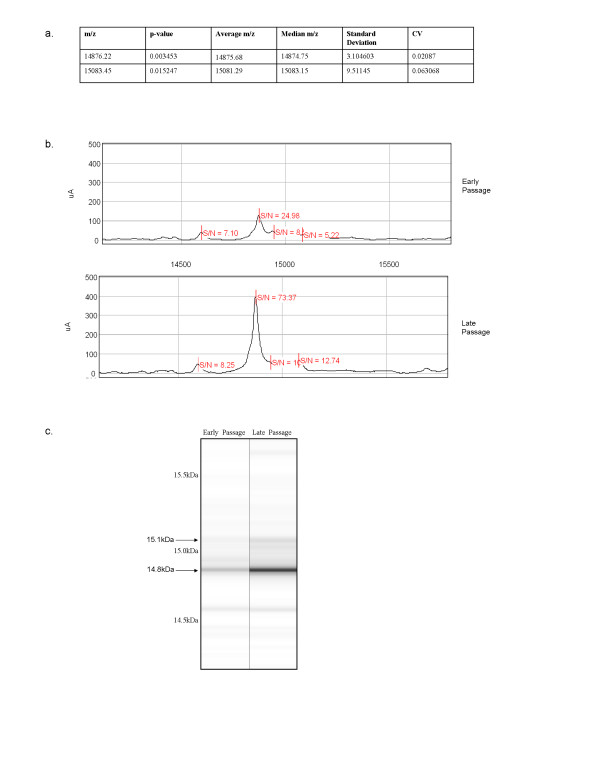
**Differentially expressed protein/peptide peaks within the region of 14.5–16.5 kDa**. Early-passage and late-passage T-cell clones were clustered into two comparative groups for analysis, for a total of 15 samples in each of the two groups, analyzed in triplicate. From the list of peak clusters created between 14.5 kDa and 16.5 kDa m/z, p-values were calculated using CiphergenExpress Client software (version 3.0.5.013). Peaks were manually scored for quality and peaks with signal:noise ratios (S/N) <5.0 in more than 50% of samples were removed from analysis. SELDI analysis identified two protein/peptide peaks that were differentially expressed between early and late passage T-cell clones. p < 0.05 was considered statistically significant. Molecular weight (Da), p-value, average mass:charge (m/z), median mass:charge (m/z), standard deviation, and CV are demonstrated for these two peaks (a). Representative early and late passage spectra (b) and gel-like view (c) are demonstrated for the region between 14.5 and 16.5 kDa.

Heat map visualisation of early and late passage T-cells identified a region between 14.5 kDa and 16.5 kDa which was highly conserved between individual samples and significantly differed between the two groups (Figure 2). In fact, two peaks within this region, of 14076 Da and 15083 Da, were found to significantly increase in expression between early and late passage cells, as shown in Figure 3. These data indicate that this region may contain protein bio-markers of immunosenescence. In light of these results, we decided to focus our attempts to identify proteins/peptides located within this size region (14.5–16.5 kDa).

### Protein identification by Nano-LC IonTrap MS/MS

**Figure 4:**
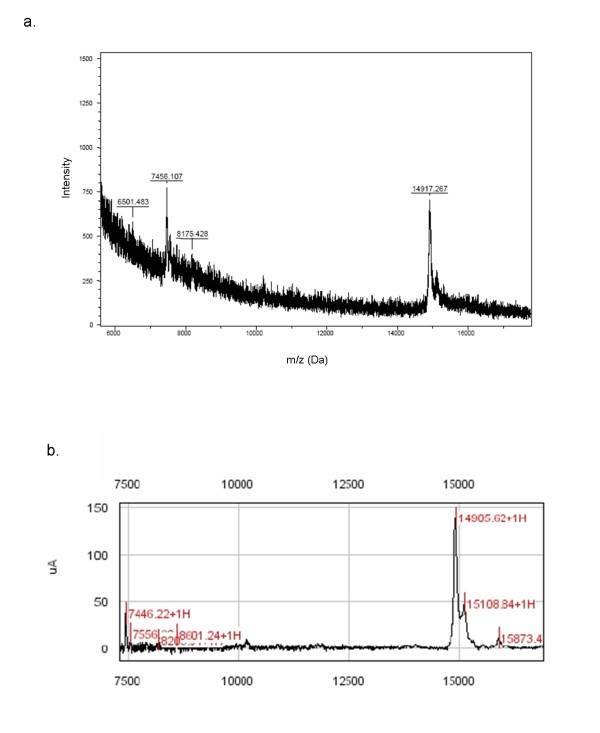
**Comparative m/z determination of the 14.9 kDa differentially expressed protein/peptide peak**. Lysates from several clones were pooled and enriched under the same reversed-phase conditions that were used in the H50 ProteinChip SELDI analysis. Reversed-phase high-performance liquid chromatography (HPLC) was performed in order to compare mass obtained from SELDI analysis to more accurate MALDI-TOF MS. HPLC-purified samples were run in parallel on SELDI-TOF (on a normal-phase ProteinChip) and MALDI-TOF mass spectrometers. One peak of 14.9 kDa identified as differentially expressed in T-cell senescence is demonstrated by MALDI-TOF (a) and SELDI-TOF (b) mass spectrometric analysis.

In order to determine the identities of the differentially expressed peaks between 14.5–16.5 kDa it was first necessary to confirm the size of the target proteins and to determine how much inter-experiment variability was introduced by SELDI analysis. For this purpose, lysates from several clones were pooled and enriched under the same reversed-phase conditions that were used in the H50 ProteinChip. Reversed-phase high-performance liquid chromatography (RP-HPLC) was performed in order to compare mass obtained from SELDI analysis to more accurate MALDI-TOF MS. RP-HPLC-purified samples were run in parallel on SELDI-TOF (on a NP20 normal-phase ProteinChip) and MALDI-TOF mass spectrometers. One peak of 14.9 kDa identified as differentially expressed in T-cell senescence is demonstrated by MALDI-TOF and SELDI-TOF mass spectrometric analysis (Figures 4A and 4B, respectively). M/z comparison of the 14.9 kDa within both samples demonstrated concordance within 20Da.

In order to identify the 14.9 kDa protein, SDS-PAGE pre-fractionation, tryptic digestion, and LC-MALDI-MS/MS of pooled T-cell clone lysates was performed. MASCOT database searching identified several different proteins from the SDS-PAGE region <25 kDa, as shown in Table 1. Several highly abundant bovine proteins were artefacts of the culture medium. However, only two human proteins, human ribosomal protein S16 and human profilin-1, were unambiguously identified within the target region of 14.5–16.5 kDa. Because of the high MOWSE score of human profilin-1 and its described role in cellular motility, survival and signalling pathways, we focussed our attempts in confirming differential expression of profilin-1 in T-cell clone senescence by Western blot analysis.

**Table 1:** Protein Identification by Nano-LC IonTrap MS/MS

**ACCESSION NUMBER**	**PROTEIN ID**	**MOWSE SCORE**	**NOMINAL MASS**
A35714	Fetuin precursor (bovine)	222	38394
ABBOS	Serum albumin precursor (bovine)	204	71221
ARF4_HUMAN	ADP-ribosylation factor 4 (human)	87	20481
COF1-HUMAN	Cofilin-1 (human)	47	18588
CSHUA	Peptidylprolyl isomerase A (human)	83	18229
R3UH16	Ribosomal protein S16 (human)	87	16549
HBBOF	Haemoglobin beta chain, fetal (bovine)	214	15963
**PROF1-HUMAN**	**Profilin-1 (human)**	**224**	**14923**
HABO	Haemoglobin alpha chain (bovine)	136	15044
H2AQ_HUMAN	Histone H2A.q (human)	63	13849
H2B_HUMAN	Histone HsB (human)	112	13752
HSHUB1	Histone H2B.1 (human)	71	13606
A48219	Calvasculin (human)	35	11721
LPHUA2	Apolipoprotein A-II precursor (human)	67	11168
LPHUC3	Apolipoprotein C-III precursor (human)	48	10846
BCHUY	Calcyclin (human)	36	10173
1C3TA	Ubiquitin mutant YES (human)	120	8560
1TBEB	Tetraubiquitin, chain B (human)	140	8176

Protein accession number, identity, MOWSE score and predicted mass (Da) are shown.

### Confirmation of differential expression of profilin-1

**Figure 5:**
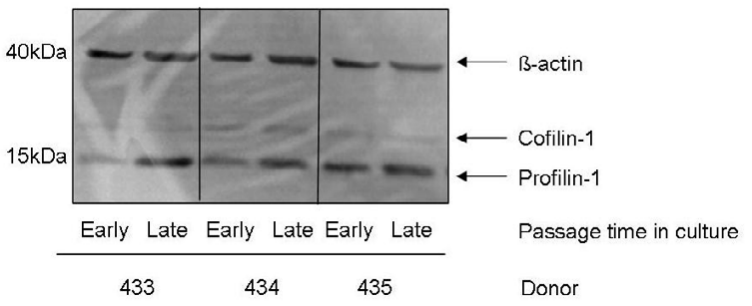
**Western blot analysis of profilin-1 expression in T-cell clones obtained from octogenarian donors**. For Western blot analysis, one million cells were pelleted and lysed in sample buffer (50 mM Tris, pH 8.0, 120 mM NaCl, 0.5% NP40, 10 μg/mL PMSF (phenylmethylsulfonylfluoride), and 1× protease inhibitor cocktail. A total of 30 μg protein was suspended in laemmli buffer (50 mM tris, pH 6.8, 1% β-mercaptoethanol, 2% sodium dodecyl sulphate, 0.1% bromophenol blue, and 10% glycerol). Samples were boiled and subject to Western blot analysis on NuPAGE™ Bis-Tris gels with MES running buffer. Human profilin-1, cofilin and β-actin proteins were detected by antibodies as detailed in *Materials and Methods *and visualised by enhanced chemi-luminescence.

In order to confirm differential expression of profilin-1 in T-cell clone senescence, proteins from early and late passage clones derived from octogenarian donors were analysed by Western blotting. A representative example of profilin-1 expression in T-cell clones derived from three octogenarian donors is shown in Figure 5. Because cofilin, a protein with opposing roles to profilin, was also identified by nano-LC IonTrap MS/MS -although it was outside the region of interest--its expression was also analysed by Western blotting. While profilin-1 expression was significantly increased in senescent T-cells, no such trend was observed for cofilin expression.

**Figure 6:**
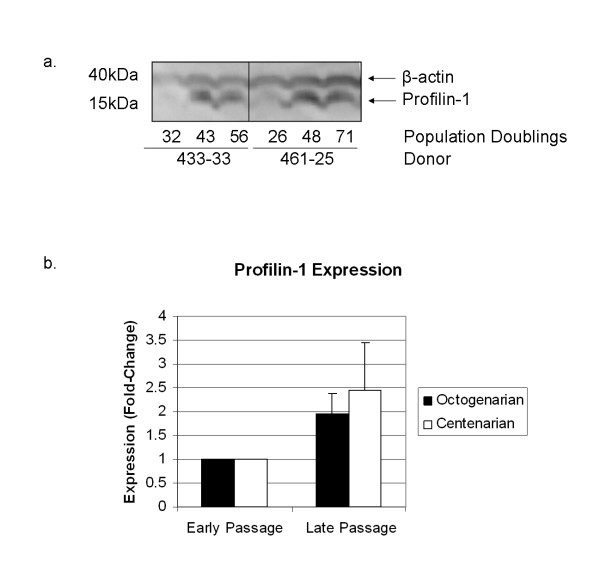
**Analysis of profilin-1 expression in T-cell clones obtained from octogenarian and centenarian donors**. For Western blot analysis, one million cells from T-cell clones obtained from octogenarian and centenarian donors at early, middle and late passage were pelleted and lysed in sample buffer (50 mM Tris, pH 8.0, 120 mM NaCl, 0.5% NP40, 10 μg/mL PMSF (phenylmethylsulfonylfluoride), and 1× protease inhibitor cocktail. A total of 30 μg protein was suspended in laemmli buffer (50 mM tris, pH 6.8, 1% B-mercaptoethanol, 2% sodium dodecyl sulphate, 0.1% bromophenol blue, and 10% glycerol). Samples were boiled and subject to Western blot analysis on NuPAGE™ Bis-Tris gels with MES running buffer. Human profilin-1 and β-actin proteins were detected and visualised by enhanced chemi-luminescence. Protein expression profiles from one representative clone from an octogenarian and centenarian donor is shown along with population doublings (PD) for each sample analysed (A). Average profilin-1 expression in early and late passage octogenarian and centenarian samples is depicted graphically following normalisation to β-actin expression in each sample (B). Student's t-test was performed and p < 0.05 was considered statistically significant.

We next investigated whether profilin expression was altered in senescent cells from other elderly donors. For this purpose T-cell clones were isolated from centenarian donors and profilin-1 expression was analysed by Western blotting, as shown in Figure 6. A representative image of profilin-1 expression in one T-cell clone from one octogenarian and one centenarian donor is depicted in Figure 6a. Average profilin-1 expression from 3–5 clones each from 3 octogenarian and 3 centenarian donors was determined at early and late passage, and is shown in Figure 6b. In agreement with data obtained from SELDI-TOF-MS analysis, profilin-1 expression is increased in senescent T-cells derived from both octogenarian and centenarian donors.

## Discussion

Clonal expansion under conditions of chronic antigenic stress is thought to contribute to the altered immune status in the elderly [27]. The accumulation of large numbers of dysfunctional, senescent cells is an important parameter associated with the "Immune Risk Phenotype" which predicts mortality in free-living elderly people [28,29]. Understanding the underlying biochemical alterations associated with altered T-cell function and the senescent phenotype is essential for developing rational intervention strategies. Therefore the purpose of the present study was to use SELDI-TOF-MS to investigate differential protein expression profiles in chronically stimulated T-cell clones as they approach senescence.

In this approach, we first detected differentially expressed proteins by SELDI-TOF-MS, confirmed the mass of these peaks by RP-HPLC and MALDI-TOF (LC-MALDI), and finally identified the proteins by nano-LC-ESI-MS/MS analysis. We focussed our efforts in this initial study to investigate proteins identified in the region of 14.5–16.5 kDa, because this area appeared to be enriched for bio-markers of immunosenescence. Advantages of the SELDI technique compared to traditional proteomic techniques include (1) ease of analysis of protein or protein conjugates in serum and patient samples, (2) functional surface chemistries allow enrichment of proteins and peptides and (3) rapid scanning and high sample throughput. While Nano-LC-ESI-MS/MS provides a higher information output than SELDI-TOF-MS, it is designed for a lower throughput of samples to be analysed. Therefore, by combining these two techniques, we maximise sample throughput while allowing heightened information output.

The majority of proteins we identified with this approach were either (1) artefacts of large bovine serum proteins present in the extract or (2) proteins outside of the range of interest, as shown in Table 1. Several factors resulted in the co-migration of bovine serum proteins with proteins of interest thereby hampering attempts to identify the latter: (1) cells were cultured in the presence of bovine-derived serum, and (2) proteins of interest were in low abundance, as evident by relatively low signal:noise ratios. However, the 14.9 kDa protein peak present in both RP-HPLC-purified and ProteinChip-enriched samples was unambiguously identified as human profilin-1 by LC-ESI-MS/MS, with a high associated MOWSE score of 224 and sequence coverage of 43%. A MASCOT database MOWSE score of 67 is associated with a p value of <0.05. Therefore, a MOWSE score of 224 gives a high degree of confidence in the identification of human profilin-1. Furthermore, the differential expression of profilin-1 was proven by Western blot analysis.

Human profilin-1 is a widely and highly expressed 14 kDa cytoplasmic protein. Profilin proteins are thought to play a central role in the regulation of *de novo *actin assembly by preventing spontaneous actin polymerisation through the binding of actin monomers and addition of monomeric actin to the barbed actin-filament ends. All stages of lymphocyte development and activation are associated with profound changes in cell morphology that depend on actin cytoskeletal remodelling. These include lymphocyte migration, homing into lymphoid organs, interaction with antigen-presenting cells, and adherence to target cells. During lymphocyte maturation, T-cell and B-cell precursors are in continuous contact with stromal cells and these physical contacts are critical for differentiation and selection. Because many signalling molecules are associated with cytoskeletal scaffolds, the cytoskeletal structure can directly regulate the molecular dynamics of signalling and biochemical responses.

The major components of the profilin-1 cytoskeleton remodelling complex are clathrin, valosine-containing protein (VCP), HSP 70, tubulin and actin [30]. It is noteworthy that VCP, which is found in the profilin-1 complex, is a major target for tyrosine phosphorylation following T-cell stimulation [31]. These data suggest that profilins may be involved in tyrosine phosphorylation signal transduction pathways. Furthermore, it was recently hypothesized that profilin may act as a negative regulator by buffering effector molecules that would otherwise be recruited and activated through SH3-domain-containing linkers [30]. In this way, profilin over-expression may lead to disrupted intra-cellular signalling and inter-cellular communication.

Efforts to define the molecular basis of cellular adhesion, underlying signal transduction pathways, and their importance in cell-cell communication and cell-matrix interactions have progressed a great deal in the last ten years. The involvement of adhesion molecules in signal transduction pathways has been well documented. Profilin is known to be physically associated with phosphatidylinositol 4,5-bisphosphate (PIP_2_) and phosphatidylinositol 3-kinase (PI-3-kinase) products and functions as a link between cytoplasmic signalling and actin assembly [23-25,32]. For example, adhesion of T-cells can be inhibited by wortmannin (an inhibitor of PI-3-kinase) and cytochalasin B (a disruptor of the actin cytoskeleton), suggesting that both G protein-sensitive PI-3-kinase and cytoskeletal assemblies are critical in the process. It has been reported that integrin-mediated adhesion of endothelial cells to fibronectin was increased by profilin over-expression and resulted in increased recruitment of fibronectin receptors to the plasma membrane. This occurred where focal contacts were being formed and co-localized with focal adhesion proteins [33]. Furthermore, profilin co-localization with spontaneously polymerized F-actin was reduced by PI 3-kinase inhibitors or expression of dominant-negative H-Ras [34]. Taken together, these data indicate that profilin-1 may play an important role in the regulation of integrin-mediated T-cell adhesion and that this function is dependent on PI-3-kinase signalling.

Several pieces of evidence suggest that PI-3-kinase signalling is impaired in ageing and senescence. Firstly, homology has been described between PI-3-kinase catalytic subunit and *Age1*, a gene influencing ageing, in *Caenorhabditis elegans *[35]. *In vitro *experiments using PI-3-kinase inhibitors have demonstrated that inhibition of PI-3-kinase signalling leads to growth retardation. Since PI-3-kinase is a major regulator of the cellular stress and survival response, it may be important to consider that altered PI-3-kinase and profilin-1 activity or expression in cellular senescence and ageing may be a response to stress and may serve to alter the cellular adhesion properties and signalling cascades.

Several of the proteins identified through the proteomics analysis reported here, namely human profilin-1, cofilin, peptidylprolyl isomerase A, cyclophilin A, and ubiquitin-related proteins have previously been identified as targets of glutathionylation in oxidatively-stressed T-cells [36]. In addition, several cytoskeletal and profilin-interacting proteins including vimentin, myosin, tropomyosin, actin, and HSP70 were also found to be targets of glutathionylation, suggesting that the actin remodelling process is tightly regulated by redox status. Protein glutathionylation may represent a means of redox regulation of protein function and may play an important role in the ageing process. Accumulation of damaged proteins in ageing has been hypothesized to be a major contributor to cellular senescence, dysfunction and disease. It remains possible that profilin-1 over-expression associated with immunosenescence might be due to activation of the cellular stress response and subsequent accumulation of oxidatively modified protein which cannot be cleared by the proteosome. This hypothesis fits with the observation that profilin-1 -and profilin-1-interacting proteins- are highly sensitive to oxidative damage. It also agrees with the documented increased levels of oxidative DNA damage found in late-passage T-cell clones [37,38]. However, to date, alterations of expression and activity of these redox-sensitive proteins, including profilin-1, in ageing remains unexplored.

In addition to the fact that several of the actin-remodelling proteins identified in the current study are targets of glutathionylation, many of these same proteins -namely peptidyl-prolyl isomerase A, cofilin and profilin-- have been demonstrated to be regulated by transforming growth factor-β (TGF-β) [39]. Because of its function as a growth factor in regulating inflammation and tissue repair, it is hypothesized that TGF-β may play a role in ageing. In fact, a number of polymorphisms within the TGF-β1 promoter have been described and are associated with longevity [40,41]. Furthermore, several groups have associated altered TGF-β activity with ageing and senescence [42-44], demonstrating a potential role for the protein in contributing to these phenotypes. In concordance with these data, we have recently found altered TGF-β signalling in senescent T-cell clones [45]. Accordingly, we propose that altered profilin-1 expression in senescence may occur in a TGF-β-dependent manner, concomitant with a variety of other signalling defects. However, further research is needed to investigate this hypothesis.

## Conclusion

In conclusion, here we report that profilin-1 expression is up-regulated in T-cell clones approaching senescence. This finding has important implications for cytoskeletal remodelling, cellular adhesion, and signalling pathways in ageing and senescence. We were unable to detect concomitant differential expression of other actin-remodelling proteins, including cofilin, suggesting that the actin polymerisation/depolymerisation homeostasis may be disrupted. Due to the described signalling role for profilin, it remains probable that altered expression could impact on a variety of biological processes including cellular survival, proliferation, motility and other as yet undescribed functions. Of interest, a recently published study has linked up-regulation of profilin-1 with retinoic-acid induced inhibition of proliferation and migration [26]. Combined with the results presented in this paper, the data strongly suggest that increased expression of profilin-1 may play a direct role in inducing the senescent phenotype. Therefore, it remains important to further investigate the biological consequences of altered profilin-1 expression in order to understand the processes underlying T-cell dysfunction that contribute to immunosenescence. Taken together, the results presented in this study highlight the discovery of a potential bio-marker of T-cell dysfunction that may have a diverse impact on cellular signalling cascades and biological processes inherent to immunosenescence.

## Methods

### Cells

For mass spectrometry, CD4+ human T-cell clones from activated peripheral blood lymphocytes of healthy octogenarian donors were obtained by limiting dilution in the presence of IL-2 as previously described [19,20]. Five representative clones were selected from each of three different octogenarian donors at time-points as early as possible in antigen-stimulated *in vitro *culture (at the first population doubling (PD) in which 2 million cells were available for analysis) and approaching senescence, at the penultimate or final PD prior to replicative senescence. These clones were not markedly different in any way from the majority of such clones that we have obtained from numerous different donors, and at isolation express markers of effector memory cells (CD45RO^++^, CD45RB^+^, CD45RA^lo^, CD28^lo^, CD95^++^, CCR7^lo^, and in addition, carry typical markers of activated T-cells (CD80, CD86, PD-L1, MHC class II). For SELDI analysis, two million cells from each clone at each time point were pelleted and resuspended in lysis buffer (H50: 8 M urea, 2% CHAPS; Q10: 50 mM Tris-HCl, 5 mM EDTA PH6.0, 2 mM PMSF, 1% Triton X-100) from T-cell clones cryopreserved at early and late passages. For Western blot analysis, between three and five T-cell clones were isolated from three octogenarian and centenarian donors as previously described [19,20] and protein expression was analysed in early and late passage cells.

### Ciphergen analysis

#### Sample preparation

A robotic automation station was utilised for automatic handling of chips including all binding and washing steps. The ProteinChip Array BioProcesessor (Ciphergen Biosystems, Inc., CA, USA) was equipped with 12 ProteinChip arrays (Ciphergen Biosystems, Inc., CA, USA), A-H format. All ProteinChip arrays were pre-treated according to manufacturer protocols. Binding buffers were 10% acetonitrile/0.1% trifluoroacetic acid (H50) and 50 mM Tris, pH 8.0 (Q10). Protein lysates from 400,000 cells (10 μl in 100 μl binding buffer) were applied to reversed-phase hydrophobic surface (H50) and strong anion exchange (Q10) surfaces. The arrays were incubated and washed according to manufacturer protocols. After the wells were dry, 1 μl saturated sinapinic acid (in 50% acetonitrile/0.5% trifluoracetic acid) was manually added to each spot. Spots were allowed to air dry and each spot was analysed in a ProteinChip Reader (Ciphergen Biosystems, Inc., CA, USA). Each sample was bound to each array surface in triplicate.

### Data acquisition and processing

ProteinChip arrays were analysed on a PCS4000 ProteinChip Reader using the ProteinChip Software version 4.0 (Ciphergen Biosystems, Inc., CA, USA). Initial arrays were read at three laser intensities before optimisation at 5000 nJ. The protocol averaged 10 laser shots per pixel with a focus mass of 18,000 Da, a matrix attenuation of 2500 Da and a range of 0–50,000 Da. The raw data were analysed in CiphergenExpress software version 3.0.5.013. The baseline was subtracted using a setting of 8 times the expected peak width, as recommended by the software developers. Ciphergen protein standard (All-IN 1 Protein Standard II, Ciphergen Biosystems, Inc., CA, USA) was analysed on an NP20 (normal phase) ProteinChip (Ciphergen Biosystems, Inc., CA, USA) using the same analysis protocol. The following peaks were identified in the resulting spectrum and used to create a three-parameter weighted internal calibration using the CiphergenExpress software (version 3.0.5.013): hirudin BKHV (7033.61 Da), bovine cytochrome c (12230.92 Da) equine cardiac myoglobin (16951.51 Da) and bovine RBC carbonic anhydrase (29023.66 Da). This internal calibration was copied and applied to the spectra as an external calibration. Data were normalised by total ion current (TIC) to an external normalisation coefficient of 0.2. The low mass cut-off was 2500 Da m/z. Normalised peaks were used exclusively in data analysis. Expression difference mapping (EDM) was utilised with automatic peak detection using the settings of five times the signal-to-noise (S/N) ratio for the first pass and two times the S/N ratio for the second pass. Peaks were detected between 2500 Da and 30,000 Da and a list of peak clusters created for each experimental sample.

### Statistical analysis

Early-passage and late-passage T-cell clones were grouped for analysis, for a total of 15 samples in each of the two groups, in triplicate. From the list of peak clusters created, p-values were calculated using CiphergenExpress software (version 3.0.5.013). Peaks were manually scored for quality and peaks with signal:noise ratios (S/N) <5.0 in more than 50% of samples were removed from analysis. Wilcoxon signed-rank tests for the matched pairs were performed and p < 0.05 was considered statistically significant.

### Confirmation of m/z by Reversed-Phase High-Performance Liquid Chromatography (RP-HPLC) with subsequent MALDI-TOF analysis (LC-MALDI)

To confirm the size of proteins of interest and to mimic the conditions of H50 Ciphergen ProteinChip, RP-HPLC fractionation on a Shimadzu Micro HPLC system was performed. 20 μl of each lysate was loaded onto a 150 × 2 mm column filled with 5 μm Reprosil C8 material (Grom, Hailfingen, Germany). A binary gradient was used, with system A consisting of 0.1% (v/v) TFA/water and system B consisting of 20% (v/v) System A/acetonitrile (ACN). At a flow rate of 350 μl/min, the gradient was run for 20 min on System A to remove the high salt content and then from 20–100 min from 0–80% System B. The eluting analytes were detected at 214 nm and 1 minute fractions were collected (350 μl). Before analysis by MALDI-TOF mass spectrometry samples were dried by lyophilisation.

Lyophilised HPLC fractions were dissolved in 50 μl HPLC grade water. 0.5 μl of each fraction were mixed with 0.5 μl saturated sinapinic acid matrix (in 50% (v/v) ACN/water with 0.1% (v/v) TFA) and crystallised on a gold MALDI target (Bruker Daltonik, Bremen, Germany). MALDI-TOF analysis was performed on a REFLEX IV™ system (Bruker Daltonik, Bremen, Germany) in the linear mode with external calibration with Protein Calibration Standard I (Bruker Daltonik, Bremen, Germany). 5 μl aliquots of the samples were used for confirmation of protein mass.

### Protein identification

Target proteins in the range below 25 kDa were isolated and identified as described recently (Tolson et al. 2006). Briefly, to reduce the complexity of the protein constituents within the lysates, SDS-PAGE was performed. 40 μg lysate was separated on a NuPAGE™ 4–12 % Bis-Tris gradient PAGE gel (Invitrogen, Karlsruhe, Germany) with MES running buffer according to the instructions of the manufacturer. Proteins were stained using the SimplyBlue™ Safe Coomassie system (Invitrogen, Karlsruhe, Germany). The entire region below approximately 25 kDa was excised into five consecutive bands, diced with a scalpel and subjected to in-gel tryptic digestion. All incubation steps were performed on a thermo-shaker and all chemicals were from Sigma (Taufkirchen, Germany) if not otherwise stated. Briefly, diced gel pieces were washed two times in 50 μl 50 mM NH_4_HCO_3_/30% acetonitrile (ACN for) 10 min at RT and then shrunk in 30 μl ACN for 5 min at RT. ACN was removed and the sample was dried in a SpeedVac for 10 min. Reduction of the proteins was performed with 5 μl 10 mM dithiothretol (DTT) in 50 mM NH_4_HCO_3_/30% ACN at 56°C for 40 min. Alkylation was performed by replacement of the DTT solution by 5 μl 40 mM iodoacetamide in 50 mM NH_4_HCO_3_/30% ACN for 30 min at RT in the dark. The gel pieces were washed two times in 50 μl 50 mM NH_4_HCO_3_/30% ACN for 10 min. After removal of the buffer, 100 μl ACN was added and the samples dried for 10 min in a SpeedVac. Digestion was performed by the addition of 250ng sequencing grade trypsin (Roche Diagnostics, Mannheim, Germany) in 50 μl 50 mM NH4HCO3 and incubation at 37°C overnight. After centrifugation (10 min 14,000 rpm) the supernatant was collected. The gel pieces were extracted 2 times with 50 μl 50% (v/v) ACN with 5% (v/v) formic acid and the extraction solutions added to the supernatant. After SpeedVac drying the samples were analysed by nanospray ionisation.

Tryptic peptides were separated on an Agilent 1100 Series HPLC system (Agilent, Waldbronn, Germany) by Nano-RP-HPLC with a 100 mm × 75 μm Grom-SIL 120 ODS-3 CP analytical column (Grom, Hailfingen, Germany). Gradient elution was performed at a flow rate of 200nl/min. The effluent of the analytical column was directly sprayed into an Esquire 3000plus™ Iontrap MS (Bruker Daltonik, Bremen, Germany) using an on-line nanoES source equipped with an 8 μm PicoTip emitter (New Objective, Woburn, MA, USA).

MS/MS data were analysed using Mascot (www.matrixscience.com) for probability-based peptide identification by database matching. The identification of each protein was evaluated based on percentage of coverage, MOWSE score (Molecular Weight Search; NCBI), number of peptide matches, peak intensity, and match of pI and molecular weight association with the SELDI-TOF-MS peaks identified as differentially expressed. Peptide and thus protein identities were considered significant when a MOWSE score greater than 35 was achieved and individual peptide fragmentation spectra showed consistent y- and b-ion series, which were assigned manually.

### Protein analysis

For western blot analysis, one million cells were pelleted and lysed in sample buffer (50 mM Tris, pH 8.0, 120 mM NaCl, 0.5% NP40, 10 μg/mL PMSF (phenylmethylsulfonylfluoride), and 1× protease inhibitor cocktail (Sigma, St Louis, USA). A total of 30 μg protein, as determined by Bradford assay (Bio-rad, Hercules, California USA) was suspended in laemmli buffer (50 mM tris, pH 6.8, 1% B-mercaptoethanol, 2% sodium dodecyl sulphate, 0.1% bromophenol blue, and 10% glycerol). Protein samples were boiled, loaded on 10% NuPAGE™ Bis-Tris gels (Invitrogen, United Kingdom) with MES running buffer according to the instructions of the manufacturer, and transferred to nitrocellulose membranes (Schleicher and Schuell, Dassel, Germany). The profilin-1 (Santa Cruz Biotechnology, USA) and cofilin (Abcam, Cambridge, UK) proteins were detected using polyclonal antibodies. β-actin protein was detected using monoclonal antibody (Abcam, Cambridge, UK). Proteins were visualised by enhanced chemi-luminescence (GE Healthcare, Buckinghamshire, UK).

## Competing interests

The author(s) declare that they have no competing interests.

## Authors' contributions

DJM drafted the manuscript and carried out the Western blot experiments and statistical analysis. GP provide cells for analysis and participated in drafting of the manuscript. RL carried out the SELDI analyses. JRP and RJF conceived the study and participated in its design. All authors read and approved the final version of the manuscript.
